# Multiple images captured from a single encounter do not promote face learning

**DOI:** 10.1177/03010066241234034

**Published:** 2024-03-07

**Authors:** Claire M. Matthews, Kay L. Ritchie, Sarah Laurence, Catherine J. Mondloch

**Affiliations:** 7497 Brock University, Canada; 7984 Toronto Metropolitan University, Canada; 4547 University of Lincoln, UK; 5488 The Open University, UK; 7497 Brock University, Canada

**Keywords:** face perception, face recognition, face learning, cognitive mechanisms, perceptual expertise

## Abstract

Viewing multiple images of a newly encountered face improves recognition of that identity in new instances. Studies examining face learning have presented high-variability (HV) images that incorporate changes that occur from moment-to-moment (e.g., head orientation and expression) and over time (e.g., lighting, hairstyle, and health). We examined whether low-variability (LV) images (i.e., images that incorporate only moment-to-moment changes) also promote generalisation of learning such that novel instances are recognised. Participants viewed a single image, six LV images, or six HV images of a target identity before being asked to recognise novel images of that identity in a face matching task (training stimuli remained visible) or a memory task (training stimuli were removed). In Experiment 1 (*n* = 71), participants indicated which image(s) in 8-image arrays belonged to the target identity. In Experiment 2 (*n* = 73), participants indicated whether sequentially presented images belonged to the target identity. Relative to the single-image condition, sensitivity to identity improved and response biases were less conservative in the HV condition; we found no evidence of generalisation of learning in the LV condition regardless of testing protocol. Our findings suggest that day-to-day variability in appearance plays an essential role in acquiring expertise with a novel face.

Recognizing facial identity is a ubiquitous task in daily life and inherently challenging. Images of two different people can be very similar, and images of the same person can vary significantly in appearance (e.g., due to changes in lighting, viewpoint, expression, makeup, health, and age) (Bindemann & Sandford, 2011; Jenkins et al., 2011; Laurence et al., 2016). The accuracy of face recognition varies as a function of familiarity. In many applied settings, employees (e.g., passport officers, clerks selling age-restricted goods) are required to match a single photograph to a live person (or a second photograph) with whom they are wholly unfamiliar. In such settings, recognition is error prone. Research participants and passport officers make errors even when images are taken only moments apart or incorporate systematic changes in lighting or expression (Duchaine & Nakayama, 2006; Megreya et al., 2013; Nordt & Weigelt, 2017; White et al., 2014).

In contrast, most people recognize familiar faces with ease, despite natural within-person variability in appearance (Jenkins et al., 2011), even when images are of poor quality (Burton et al., 1999), have been distorted (Hole et al., 2002), or when the face is disguised (Noyes & Jenkins, 2019). Recognizing highly familiar faces (e.g., friends, family, and actors) allows people to navigate social interactions or follow the plot of a movie. The effect of familiarity can be indexed by event-related potentials (ERPs). Both the N250 and the sustained familiarity effect (400–600 ms after stimulus presentation) are larger when participants view personally familiar as compared to wholly unfamiliar faces (Wiese et al., 2019).

In other settings, we might attempt to recognize a face that is neither wholly unfamiliar nor highly familiar. Such faces might have been encountered only once or only in a single setting. For example, people might need to recognize their waiter in a restaurant, someone with whom they briefly interacted at a party, their lecturer, or the perpetrator of a crime they witnessed. These lesser-known faces might be challenging to recognize for several reasons including short exposure time, sparse social representation, or minimal exposure to within-person variability in appearance (Devue et al., 2019; Ritchie & Burton, 2017; Schwartz & Yovel, 2019; Shoham et al., 2021). This middling level of familiarity is evident in ERPs. Wiese et al. (2019) reported comparable differences between the amplitude of the N250 for lecturer or celebrity faces as compared to unfamiliar faces, and a weaker sustained familiarity effect for lecturer and celebrity faces as compared to personally familiar faces.

In the current study we examined whether people recognize new instances of a person's face after a single encounter (i.e., after viewing moment-to-moment changes in head orientation, facial expression and facial speech but in the absence of variability in capture conditions, such as lighting, and changes in appearance that occur over time, such as hairstyle, make-up and health.) Understanding the process by which a newly encountered face becomes familiar is important, both for models of face recognition and in applied settings. Raviv et al. (2022) note that learning has been defined in two different ways. The first definition refers only to the ability to recognize items from a training phase (e.g., a child learning that their family pet is labelled “dog”). Traditional approaches to studying face recognition have adopted this definition of learning by presenting the same (or nearly identical) image of to-be-learned identities at study and test. For example, studies have shown that viewing the same face image from multiple viewpoints facilitates recognition of the face from those viewpoints (Hill et al., 1997; Troje & Bülthoff, 1996; Wallraven et al., 2002). The second definition defines learning as the ability to generalize from items presented during a training phase to new instances (e.g., a child learning that poodles, spaniels, and Australian shepherds are all dogs). We adopt this definition in the current study as it better captures how we recognize faces in daily life (see Burton, 2013 for a review). Familiarity allows perceivers to generalize beyond previous experience with a face (e.g., photos presented during a study phase; previous encounters with a live person) such that new instances of that face are recognised.

Exposure to within-person variability in appearance plays a key role in face learning. Viewing multiple images of an identity improves performance on memory tasks in which participants must decide whether test images belong to a previously learned identity (Baker & Mondloch, 2019; Matthews & Mondloch, 2018b; Murphy et al., 2015; Zhou et al., 2022), and on line-up tasks—at least on target-present trials (Dowsett et al., 2016; Matthews & Mondloch, 2018a). However, the evidence for whether variability helps with face matching tasks in which participants must decide whether two or more test images belong to the same person or two different people is mixed (see Matthews & Mondloch, 2018b; White et al., 2014; but see Menon et al., 2015; Ritchie et al., 2021, 2020). Whereas learning tasks require memory, matching tasks present all the images simultaneously with no memory requirement. Ritchie et al. (2021) demonstrated that memory demands may be key to a role for variability. When an array of images showing within-person variability was displayed prior to a test image, variability aided performance. When the array and test image were presented simultaneously, variability led to a shift in response bias with no improvement in accuracy. The authors argued that tasks requiring memory force participants to abstract a representation of the person from the variable images in order to compare a subsequent image to that representation. This evidence aligns with theoretical models which suggest that exposure to within-person variability in appearance allows the perceiver to form a representation that contains multiples instances of a person's appearance and/or an average of all encountered instances—a representation that allows generalisation to new instances (Burton et al., 2005, 2016; Kramer et al., 2015; Young & Burton, 2017).

The above findings are consistent with evidence from other domains in which exposure to variability during learning (i.e., the process of building a representation) leads to generalization. For example, viewing dangerous items from multiple categories (e.g., guns, knives) improves the ability to detect novel dangerous items in luggage (Gonzalez & Madhavan, 2011). Similar results have been reported for texture discrimination, language learning, and motor skills and concept formation (Hussain et al., 2012; Watson et al., 2014; see Raviv et al., 2022 for a theoretical review). Exposure to high variability (e.g., multiple speakers in a word learning task) may initially impair learning, but ultimately improves the ability to generalize (e.g., to recognize a newly learned word when pronounced by novel speakers). Across domains, variability has been characterised in different ways. One type of variability defined by Raviv et al. is heterogeneity. Heterogeneity refers to how variable examples are during learning (i.e., during training). In the case of face learning, heterogeneity refers to variability in the appearance of a to-be-learned face during the learning/study phase. Controlling for numerosity (another type of variability, i.e., the number of images of the to-be-learned face presented during learning), heterogeneity might benefit recognition because it provides greater coverage of the identity's appearance, allowing generalisation to previously unseen exemplars. In the current study, we examined the role of heterogeneity in face learning.

Studies that use multiple images to aid in learning almost always incorporate images that were captured on different days—images that serve as a proxy for high variability images (e.g., Andrews et al., 2015; Dowsett et al., 2016; Matthews & Mondloch, 2018a; White et al., 2014). Such images capture changes that occur over time (e.g., changes in hairstyle, makeup, age, weight, health), changes in capture conditions (e.g., camera lens, lighting, context), and changes that occur from moment to moment (e.g., different facial expressions, viewpoints). Of course, the degree of variability captured by such images will vary across identities. Watching the nightly news will provide less variable input from the anchor, who sits at the same desk portraying a similar appearance from night to night, than the reporter, who appears in a wide range of conditions. Nonetheless, images captured on different days can capture variability not available in a single encounter. In the current study, we examined whether adults benefit from images that were captured on the same day, under the same lighting conditions and with the same camera—images we will refer to as low-variability (LV) images. LV images capture moment-to-moment changes (e.g., changes in expression and viewpoint), but not changes that occur over time.

Ritchie and Burton (2017) examined the role of heterogeneity in face learning by directly comparing participants’ performance on a memory task after learning from either 10 images captured on different days (high variability) or 10 images captured all on the same day (low variability). Participants were more accurate on a name verification task in the high- as compared to the LV condition. These results suggest that exposure to images captured on a single encounter promotes less generalisation of learning than images captured on different encounters. However, Ritchie and Burton did not include a control group, so it is unclear whether viewing LV images provided any benefit. In a second experiment, participants completed a matching task after learning the identities from either high-variability (HV) or LV images. Performance in these two conditions was compared with a control group who had not viewed any learning images. Learning from HV images resulted in more accurate performance on match trials (i.e., those that included two images of the same person) than learning from LV images but learning from LV images produced no benefit over the control group.

Two additional studies suggest that exposure to low variability in appearance might not facilitate generalisation of learning, even when test stimuli were captured on the same day as stimuli presented in the learning phase, requiring minimal generalisation. Chen and Liu (2009) examined face learning from viewing systemic changes in viewpoints and emotional expressions captured on a single day. Training participants on multiple different poses of an identity led to better recognition of that identity across different expressions, but training on several expressions did not improve recognition across poses. Likewise, Lander and Bruce (2003) found no advantage for viewing multiple images extracted from a single video over viewing a single image, even when participants were tested with novel images of the learned identities taken on the same day (see also Matthews et al., 2007).

Results from one study suggest that exposure to low variability in appearance might be sufficient to facilitate adults’ learning of a newly encountered face in novel instances (Baker et al., 2017). Baker and colleagues compared learning after viewing 10 images extracted from a video filmed across 3 days (high variability) vs. a single day (low variability). Adults showed evidence of learning in both the HV and LV conditions relative to a no-training control group, with no difference between the HV and LV conditions. It is noteworthy that a reference photo of the target was provided to participants while they completed the old/new recognition task. The reference image was taken from a different source; thus, even in the LV condition, participants had exposure to between-day variability in appearance, albeit minimal. It is possible that the reference photo helped participants decide if the test images belonged to the identity, perhaps by alerting perceivers to potential variability in appearance.

One previous study reported no evidence that high variability in training stimuli facilitates generalisation of face learning. Honig et al. (2022) showed participants three images of a to-be-learned face previously judged as very similar to one another (low variability) or very dissimilar (high variability). In a subsequent face matching task, there was no benefit of viewing HV images during the study phase. Indeed, an advantage was observed for viewing LV images when test images were similar to those images. Honig et al. conclude that any benefit of highly variable images is attributable to their similarity to test images; the odds of a test image being similar to any study image are higher if study images are highly variable. It is noteworthy that Honig et al. only presented three learning images; as noted by Raviv et al. (2022), variability can hinder learning initially, but benefit generalisation as learning proceeds. Further, in the field of face recognition, the very essence of familiarity is the ability to recognize instances that are very dissimilar to previous encounters (e.g., when a neighbour is sick, after aging, when a colleague changes their hair or make-up). Nonetheless, further examination of how variability shapes face learning is needed.

In the current study, we examined the extent to which viewing different images from a single encounter leads to face learning, and whether the ability to benefit from viewing images from a single versus multiple encounter(s) depends on whether the task requires face matching versus face memory. In each of two experiments, participants learned one identity in each of three conditions: six HV images (captured across six days), six LV images (captured on a single day) and a single image. We included a single-image condition to allow us to examine whether viewing LV images provides any significant benefit over viewing only one image. After each learning phase, participants completed a task in which they needed to recognise novel images of the learned identity intermixed with images of other people. All test images were taken on different days than the study images, allowing us to examine generalisation of learning. In Experiment 1, participants were asked to select all the images of the target from four 8-image arrays; in Experiment 2, participants were asked to decide whether sequentially presented images belonged to the target. Based on an abundance of previous research, we expected better performance in the HV condition as compared to the control condition. Our key question was whether there was any evidence of generalisation of learning in the LV condition. If exposure to LV images is sufficient to facilitate learning, we would expect to see a benefit of viewing LV images relative to only viewing a single image. If exposure to LV images is not sufficient to facilitate learning, we would expect to see comparable performance in the LV and control conditions. The HV condition was included to determine whether any learning observed in the LV condition matched that observed in the HV condition.

In both experiments, we manipulated whether the images remained visible (face matching condition) or were removed (face memory condition) while participants completed the test phase. Some studies have suggested that the benefit of exposure to variability is only present in tasks requiring memory in which the training stimuli are removed from view before participants are asked to provide a response (e.g., Menon et al., 2015; Ritchie et al., 2021; Sandford & Ritchie, 2021). Whereas simultaneous tasks allow the perceiver to perceptually match images, sequential tasks require the perceiver to form a robust representation of each identity in memory and use that representation to recognise identities when the images are no longer visible. Extracting a robust mental representation of the variability in appearance might be necessary for learning—in which case any benefit of viewing HV or LV images might only be seen in the memory condition.

## Experiment 1

### Method

Each participant learned three identities, one in each of three learning conditions: a 1-image condition, a LV condition, and a HV condition. To assess generalisation of learning, participants completed a line-up task in which they were asked to select all the images of a target identity from each of four 8-image arrays. Half of the participants completed a matching task (i.e., the training stimuli remained visible during the recognition task) and half completed a memory task (i.e., the training stimuli were not visible during the recognition task).

#### Participants

The sample comprised 71 young adults (57 female; *M*_age_ = 19.59 years, *SD* = 2.99, range = 17–37) recruited through the online psychology research pool at Brock University in Canada. One additional participant was excluded for experimenter error. A power analysis using GPower software (Version 3.1.9.4; Faul et al., 2007) indicated that this sample was sufficient to detect a medium effect for any interactions with 99% power (α = .05). All participants provided written informed consent and were compensated with research participant credit.

#### Materials

Images from Ritchie and Burton's (2017) study were used in this task. Three female White Australian celebrities served as target identities. We confirmed that all participants were unfamiliar with these identities. All images were cropped to 400 by 300 pixels (72 ppi), and any background information was removed. All images were presented in colour.

##### Training Stimuli

**Figure 1.**
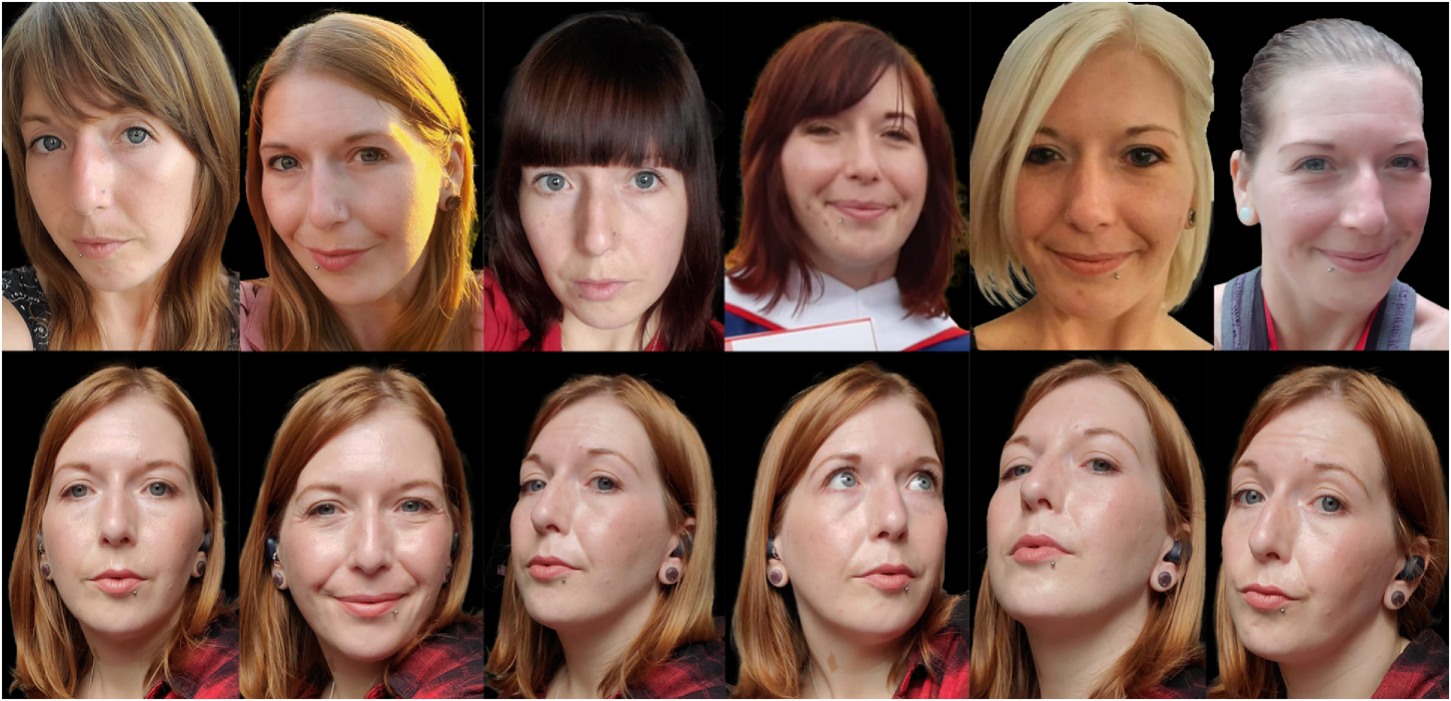
An example of the training stimuli used in the high-variability condition (top row) and the low-variability condition (bottom row). All of these images depict the same person. Copyright restrictions prevent publication of the actual images used.

We created two sets of training stimuli for each target identity, each of which comprised six images. The two sets differed in heterogeneity (see Figure 1). Images for the HV condition were obtained through Google Search. For each identity, we selected six images that were captured on different days and met the following criteria: the face was at least 150 pixels in height, was shown from a mostly frontal view and was free from occlusions. These unconstrained images included changes in appearance from day-to-day (e.g., lighting, hair style, and makeup) and moment-to-moment (e.g., facial expression and viewpoint). Images for the LV condition were taken from an interview of each target identity that was obtained through Google Video Search. For each celebrity we selected six still images captured from a single video. Like the HV images, the LV images captured moment-to-moment changes in head orientation and facial expressions; they also included variability in appearance associated with facial speech. Unlike the HV images, the LV images did not include day-to-day changes in appearance (e.g., changes in hairstyle, health, and make-up). The quality of the HV and LV images was comparable. For the 1-image control condition, a single image was selected from the six HV images; the selection of this image was counterbalanced across participants. All training images were printed on cards measuring 5.5 cm in height and backgrounds were removed.

##### Recognition Task Stimuli

Ten additional images of each target were used as test stimuli in the recognition task; all were taken on a different day from each other and from the training stimuli. The 10 target images were intermixed with images of 10 similar-looking distractors (i.e., similar age, hair colour, and face shape). We presented one image of each distractor; distractor images were selected using the same criteria as for target images. These 20 test images were used for our main analyses.

We also included control stimuli. Specifically, we captured four new images from the video used to create the LV training stimuli. We used these LV control stimuli to verify that participants in the LV condition recognised new same-day images. This was important to establish that participants had attended to the learning images and that any errors on test stimuli reflected poor sensitivity to identity when tested with images that required generalizing beyond the type of variability encountered during learning. Eight other images were used as exclusion criteria; they were designed to verify that participants paid attention and understood the task. These attention checks included four images identical to those presented in the training phase and four images of a dissimilar looking identity (i.e., different age, hair colour, and face shape). In the LV and HV conditions, attention check images of the learned identity were randomly selected from those shown during learning; in the 1-image condition, the same previously viewed learning image was shown four times. To pass the attention checks, participants needed to correctly identify at least three of the four previously viewed images in each condition and correctly reject all four dissimilar distractor images in each condition.

#### Procedure

All procedures received clearance from the research ethics board at Brock University. Each participant learned three identities, one in each of three conditions. The assignment of target identities to condition and the order in which the conditions were presented was counterbalanced across participants. The procedure was adapted from Matthews et al. (2018). In the learning phase, participants were read a short story about a target identity that embarks on an adventure. Participants viewed the six HV images in the HV condition, and the six LV images in the low variability condition. Each of the six pages in the storybook contained a new image of the target attached to the page with Velcro. Before moving to the next page, participants detached the photo and placed it on a cardboard stand. Participants were told they would need these photos to perform a later task. In the 1-image condition, participants viewed only one of the six HV images. The selection of this image was counterbalanced across participants. Participants moved the single image through each page of the story book and placed it on a cardboard stand at the end of the story. At the end of each story, participants were asked to take a moment to look at the images they collected before proceeding to the recognition task.

The recognition task was presented to participants on an Apple iPad (2048 by 1536 pixel display; 264 ppi). Participants were presented with four 8-image arrays. Arrays were presented sequentially. Each array contained two attention checks (a previously viewed training image and a dissimilar distractor), a LV control stimulus (a novel image captured from the same video as the LV training stimuli) and five test stimuli. Test stimuli comprised novel images of the target identity and images of a similar-looking distractor. Participants were explicitly told that the number of images of the target identity could vary across arrays; in fact, the number of target images ranged from one to four (e.g., one image of the target and four images of the distractor, two images of the target and three images of the distractor). This was done to reduce response bias (e.g., prevent participants from always selecting the one best image from each line-up). The images within each array were randomly selected and remained the same for all participants. The position of each image within the array and the order in which the four arrays were presented were randomised across participants. For each array, participants were asked to select all the images of the target identity before tapping a button to proceed to the next array. Participants were not given a time limit for making their selection(s).

Half of the participants (*n* = 34) performed the face matching version of the recognition task, and the other half of participants (*n* = 35) performed the face memory version. In the matching condition, the images collected during the learning phase remained visible during the recognition task. In the memory condition, the learning images were removed before participants did the task. Once the recognition task was complete, participants were introduced to the next target identity and the procedure was repeated.

### Results and Discussion

#### Primary Analyses

Our primary analyses included the 20 test stimuli (10 novel images of the target identity; 10 images of the distractor) to examine the effects of variability in training images and task structure (matching vs. memory) on sensitivity to facial identity. We defined a hit as selecting an image of the target identity and a false alarm as selecting an image of the distractor; across the four trials, a maximum of 10 hits and 10 false alarms were possible. We conducted separate 2 (Task: Matching/Memory) by 3 (Variability: 1-image/Low/High) mixed ANOVAs for d′ and criterion. Higher d′ values indicate greater sensitivity to identity, and higher criterion values indicate a more conservative response bias (i.e., fewer hits and fewer false alarms).^1^ All tests were two-tailed. We used the Bonferroni correction to correct for multiple comparisons when analysing pairwise comparisons. In addition to traditional frequentist hypothesis testing, we included Bayes factors using JASP Version 0.14.01 (JASP Team, 2020), which allowed us to quantify the extent to which the data support the alternative hypothesis (BF_10_). For reference, BF > 10 indicates decisive evidence for the alternative hypothesis; BF10 > 3 indicates substantial evidence and BF10 > 1 indicates anecdotal evidence. BF10 < .33 and BF10 < .1 indicate substantial and anecdotal evidence for the null hypothesis, respectively (Wetzels et al., 2011). The data and analysis syntax are available at: https://osf.io/mh97y/?view_only=0a3c20b10aec4737ad5d1097f0a6f5b5.

#### *d*′

**Figure 2.**
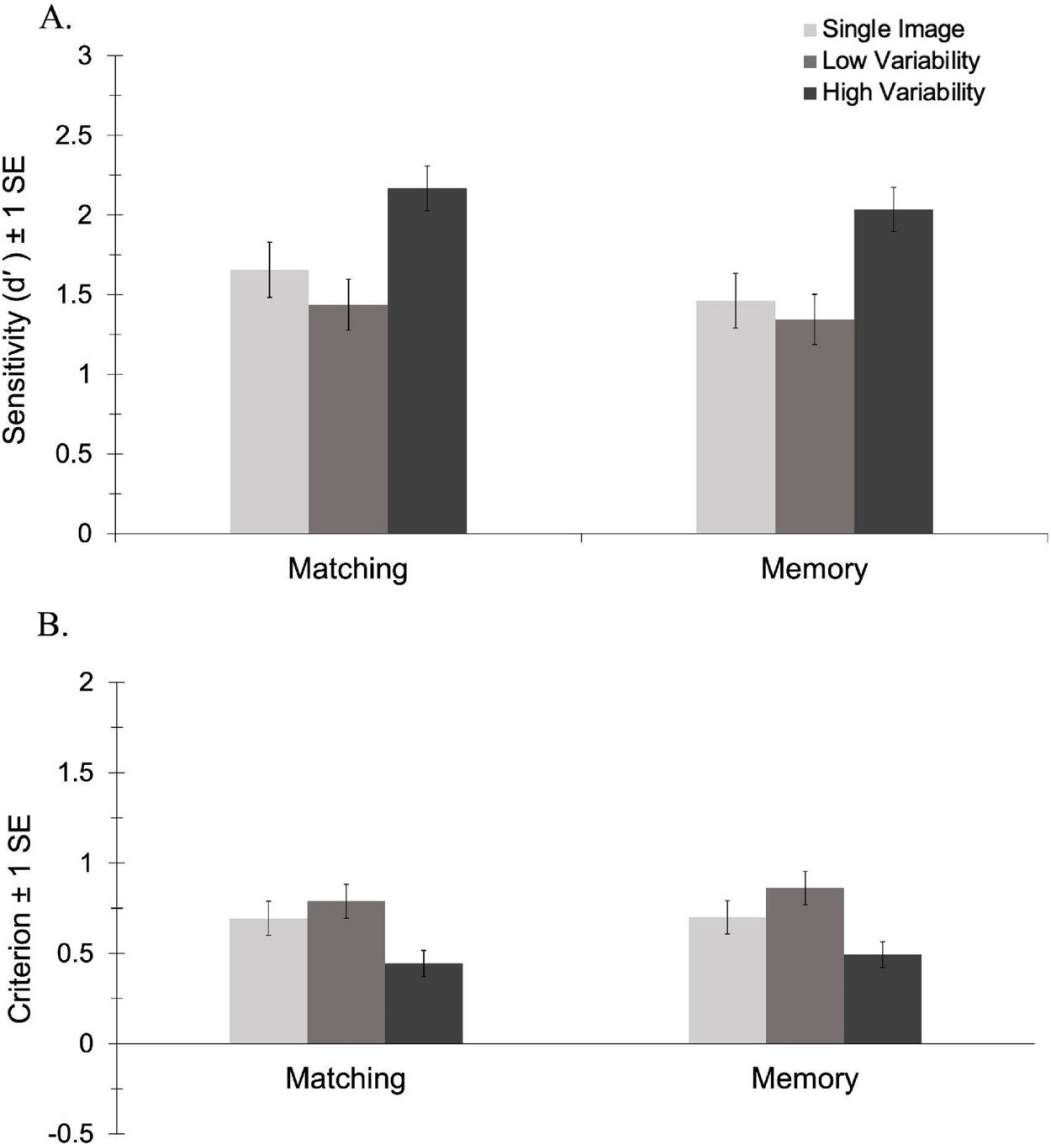
Sensitivity (A) and criterion (B) by variability for the matching and memory conditions in Experiment 1.

The analysis of *d*′ revealed no significant main effect of task, *F*(1, 69) = 0.59, *p* = .444, *η_p_*^2^ = .01, BF_10_ = 0.24 and no interaction between task and variability, *F*(2, 138) = 0.10, *p* = .901, *η_p_*^2^ < .01, BF_10_ = 0.10. There was a main effect of variability, *F*(2, 138) = 21.95, *p* < .001, *η_p_*^2^ = .24, BF_10_ = 2.042 × 10^6^. Consistent with previous studies, pairwise comparisons revealed that participants were more sensitive to identity in the HV condition (*M* = 2.10, *SE* = 0.10) than in both the LV (*M* = 1.39, *SE* = 0.11), *p* < .001, BF_10_ = 1.270 × 10^6^, and the 1-image conditions (*M* = 1.56, *SE* = 0.12), *p* < .001, BF_10_ = 1.381 × 10^3^. As shown in Figure 2A, there was no difference in sensitivity between the low variability and 1-image conditions, *p* = .391, BF_10_ = 0.40. This novel finding suggests that only exposure to high variability in appearance facilitated learning. Viewing 10 different images of an identity taken within a single encounter conferred no benefit on subsequent recognition—an effect that did not differ in the matching vs. memory versions of the task.

#### Criterion

The analysis of criterion revealed no significant main effect of task, *F*(1, 69) = 0.17, *p* = .682, *η_p_*^2^ < .01, BF_10_ = 0.25 and no interaction between task and variability, *F*(2, 138) = 0.19, *p* = .827, *η_p_*^2^ < .01, BF_10_ = 0.11. There was a main effect of variability, *F*(2, 138) = 20.38, *p* < .001, *η_p_*^2^ = .23, BF_10_ = 5.768 × 10^5^. Pairwise comparisons revealed that participants had a less conservative response bias in the HV condition (*M* = 0.47, *SE* = 0.05) than in both the LV (*M* = 0.83, *SE* = 0.07), *p* < .001, BF_10_ = 5.901 × 10^6^, and the 1-image conditions (*M* = 0.70, *SE* = 0.07), *p* < .001, BF_10_ = 145.16. As shown in Figure 2B, there was no difference in criterion between the low variability and 1-image conditions, *p* = .109, BF_10_ = 1.14. This novel finding suggests that exposure to high, but not low, variability in appearance shifts perceivers’ decision boundary such that more novel images are perceived as belonging to the newly learned identity.

#### Low Variability Control Images

We conducted a 2 (Task: Matching/Memory) by 3 (Variability: 1-image/Low/High) mixed ANOVA with the number of recognised new low variability images as the dependent variable. The analysis revealed no significant main effect of task, *F*(1, 69) = 0.00, *p* = .999, *η_p_*^2^ < .01, BF_10_ = 0.15, and no interaction between task and variability, *F*(2, 138) = 0.01, *p* = .988, *η_p_*^2^ < .01, BF_10_ = 0.06. There was a main effect of variability, *F*(2, 138) = 47.54, *p* < .001, *η_p_*^2^ = .41, BF_10_ = 3.00 × 10^15^. Pairwise comparisons confirmed that participants in the LV condition recognised more LV images (*M* = 3.99, *SE* = 0.01) than participants in both the HV (*M* = 2.90, *SE* = 0.17) *p* < .001, BF_10_ = 1.55 × 10^6^, and 1-image conditions (*M* = 2.14, *SE* = 0.20) *p* < .001, BF_10_ = 3.51 × 10^11^. Participants also recognised more LV images in the HV condition than in the 1-image condition, *p* = .002, BF_10_ = 50.77. This suggests that participants were attending to LV images; nonetheless, these images only facilitated recognition of the identity in new images that were captured on the same day as the trained images. It is noteworthy that facilitation of recognition of images taken on the same day occurred despite background information being removed during the study phase.

Our results reveal that only exposure to HV images led to generalisation of learning, such that participants’ sensitivity to identity in novel images increased. Viewing high-variability images led participants to adopt a less conservative response bias relative to viewing a single image. Viewing LV images only provided a benefit for new images that were taken from the same encounter as the learned images, suggesting that exposure to changes in appearance that occur over time is necessary to facilitate recognition of novel images taken on different days (i.e., generalisation of learning).

In Experiment 1, we used a line-up paradigm in the test phase. In this type of task, participants can make relative judgements about the images (i.e., compare to the other test images in the line-up when deciding if an image belongs to the target). In Baker et al. (2017), in which evidence of learning from low-variability images was reported, participants completed a Yes/No task in which they viewed images individually and were asked to decide whether each image belonged to the target. This type of test involves a more absolute judgement about each image than the line-up task (i.e., participants had to decide if each image was an image of the target without comparing it to any other test images). To examine whether this difference in testing protocols might have contributed to our finding no evidence of learning in the LV condition, we changed our testing task in Experiment 2 to mimic Baker et al.'s task. Participants completed the same learning protocol but instead of the test images being presented in a line-up, each image was presented sequentially.

## Experiment 2

### Method

#### Participants

The sample comprised 73 young adults (54 females; *M*_age_ = 19.87 years, *SD* = 3.46, Range = 17–38) recruited through the online psychology research pool at Brock University in Canada. Six additional participants were excluded (five for experimenter error and one for failing attention checks). A power analysis using GPower software (Faul et al., 2007) indicated that this sample was sufficient to detect a medium effect for any interactions with 99% power (α = .05). All participants provided written informed consent and were compensated with research participant credit.

#### Materials and Procedure

The materials and procedure were identical to Experiment 1, except for the presentation of images in the recognition task. Here, images were presented one at a time on the screen in a randomised order (32 trials). Participants were asked to decide whether each image depicted the target identity. Participants responded by pressing a button on the touch screen of the iPad. There was no time limit for viewing each image. As in Experiment 1, half of the participants (*n* = 36) performed a matching version of the recognition task where the training images remained visible, and the other half of participants (*n* = 37) performed a memory version where the training images were taken away before the recognition task.

### Results and Discussion

All analyses were identical to those in Experiment 1. When Mauchly's test of sphericity indicated that the assumption of sphericity was violated, the degrees of freedom were adjusted using the Greenhouse–Geisser correction. The data and analysis syntax are available at: https://osf.io/mh97y/?view_only = 0a3c20b10aec4737ad5d1097f0a6f5b5.

#### *d*′

**Figure 3.**
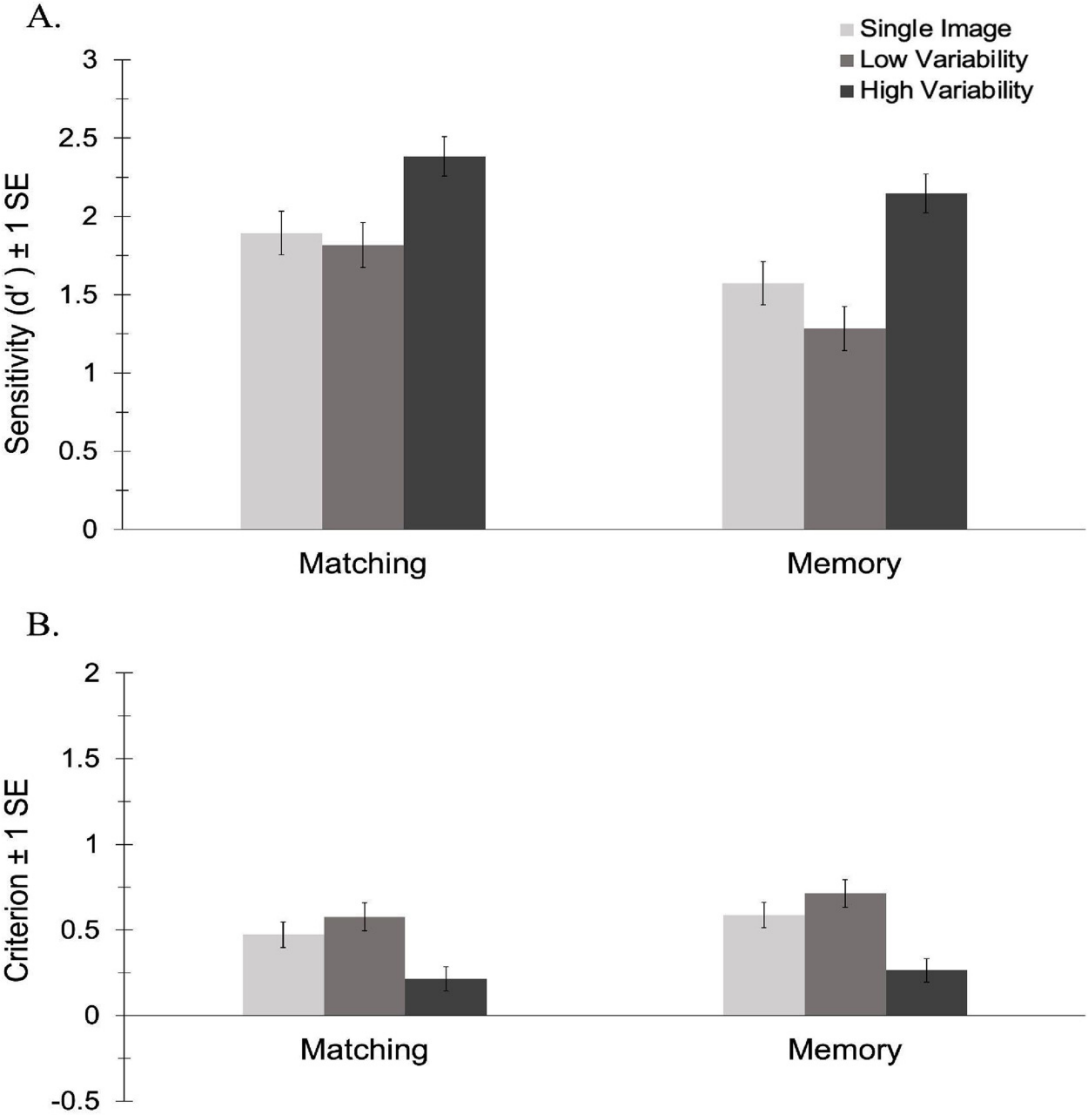
Sensitivity (A) and criterion (B) by variability for the matching and memory conditions in Experiment 2.

The analysis of *d*′ revealed a significant main effect of task, *F*(1, 71) = 5.70, *p* = .20, *η_p_*^2^ = .07, BF_10_ = 2.24. Participants were more sensitive to identity in the matching task (*M* = 2.03, *SE* = 0.11) than in the memory task (*M* = 1.67, *SE* = 0.11). There was a main effect of variability, *F*(2, 142) = 27.06, *p* < .001, *η_p_*^2^ = .28, BF_10_ = 7.908 × 10^7^. As predicted, pairwise comparisons revealed that participants were more sensitive to identity in the HV condition (*M* = 2.14, *SE* = 0.10) than in both the LV (*M* = 1.41, *SE* = 0.12), *p* < .001, BF_10_ = 5.381 × 10^7^, and the 1-image conditions (*M* = 1.61, *SE* = 0.12), *p* < .001, BF_10_ = 1.656 × 10^4^. There was no significant difference between the low variability and 1-image conditions, *p* = .277, BF_10_ = 0.52. As shown in Figure 3A, there was no interaction between task and variability, *F*(2, 138) = 1.16, *p* = .317, *η_p_*^2^ = .02, BF_10_ = 0.64. This suggests that the effect of variability does not differ as a function of task. As in Experiment 1, only exposure to high variability in appearance facilitated learning and this effect did not differ in the matching vs. memory versions of the task.

#### Criterion

The analysis of criterion revealed no significant main effect of task, *F*(1, 71) = 1.40, *p* = .241, *η_p_*^2^ = .02, BF_10_ = 0.37, and no interaction between task and variability, *F*(1.81, 128.58) = 0.34, *p* = .694, *η_p_*^2^ = .01, BF_10_ = 0.14. There was a main effect of variability, *F*(1.81, 128.58) = 28.57, *p* < .001, *η_p_*^2^ = .29, BF_10_ = 2.451 × 10^8^. Pairwise comparisons revealed that participants had a less conservative response bias in the HV condition (*M* = 0.24, *SE* = 0.05) than in both the LV (*M* = 0.65, *SE* = 0.06), *p* < .001, BF_10_ = 1.367 × 10^6^, and the 1-image condition (*M* = 0.53, *SE* = 0.05), *p* < .001, BF_10_ = 3.970 × 10^5^ As shown in Figure 3B, there was no significant difference in criterion between the low variability and 1-image conditions, *p* = .118, BF_10_ = 1.05—a pattern that mimics the results of Experiment 1.

#### Low Variability Control Images

A 2 (Task: Matching/Memory) by 3 (Variability: 1-image/Low/High) mixed ANOVA, with the number of LV control images recognised as the outcome, revealed no significant main effect of task *F*(1, 71) = 0.531, *p* = .469, *η_p_*^2^ = .01, BF_10_ = 0.18 and no interaction between task and variability, *F* (1.68, 119.31) = 1.84, *p* = .302, *η_p_^2^* = .02, BF_10_ = 0.17. There was a main effect of variability, *F* (1.68, 119.31) = 33.77, *p* < .001, *η_p_*^2^ = .32, BF_10_ = 1.584 × 10^11^. Pairwise comparisons revealed that participants in the LV condition recognised more LV control images (*M* = 3.99, *SE* = 0.01) than did participants in both the HV (*M* = 3.11, *SE* = 0.15) *p* < .001, BF_10_ = 1.022 × 10^5^, and 1-image conditions (*M* = 2.44, *SE* = 0.19) *p* < .001, BF_10_ = 2.185 × 10^9^. Participants in the HV condition recognised more LV control images than did participants in the 1-image condition, *p* = .011, BF_10_ = 7.86. As in Experiment 1, this suggests that exposure to LV images facilitates learning for new images taken from the same encounter but does not allow generalisation of learning for new images outside that range of variability.

The results of Experiment 2 demonstrate that exposure to images captured on different days increased sensitivity to the target identity and led participants to adopt a less conservative response bias; exposure to images captured on a single day did not. Indeed, any numerical differences in the LV condition relative to the single-image condition were in the opposite direction. Viewing LV images during learning improved recognition of other images taken on the same day, relative to viewing either a single image or six images taken on different days. Collectively, the results of Experiments 1 and 2 suggest that in both a Line-up and Yes/No task, regardless of whether the learning images were taken away or displayed during test, only exposure to high variability in appearance facilitated generalisation of learning to novel images taken on different days.

## General Discussion

Several studies have demonstrated that exposure to within-person variability facilitates face learning, such that increased sensitivity to identity generalizes to new images taken on different days. Abundant research has provided evidence of learning after participants view multiple images of a target identity that incorporate variability in appearance over time (Andrews et al., 2015; Dowsett et al., 2016; Matthews & Mondloch, 2018a; Ritchie & Burton, 2017; White et al., 2014). In the current study, we investigated whether adults also show evidence of generalisation when viewing images that were captured from a single encounter. We compared participants’ face learning after viewing either a single image, six LV images or six HV images in both a matching and a memory task using two different testing paradigms. Across all these conditions, we found no evidence of generalisation after viewing LV images. Only exposure to HV images increased sensitivity for the target identity in novel instances and shifted response bias.

Our findings of a benefit of viewing high vs. low variability are consistent with Ritchie and Burton (2017), but the original study did not include the single image control condition that we included here. We have extended the Ritchie and Burton (2017) finding to show that, in fact, generalisation of learning does not occur through viewing a set of images captured in a single encounter. The only advantage for viewing such low variability images in Ritchie and Burton's study was in faster response times when test images showed new images from the same LV set as the learning images, a pattern akin to that reported by Honig et al. (2022). This shows that participants paid attention to low variability images and so rules out a simple attention-based explanation for the lack of generalisation when tested with HV images. Our findings complement and extend Lander and Bruce (2003) who found no learning advantage for viewing multiple images extracted from a single video over viewing a single image.

Participants showed a low variability advantage with test images taken from the same encounter. This finding suggests that similarity between learning and test images may play a role in face learning. Evidence for the importance of similarity was shown by Sandford and Ritchie (2021) who used HV and LV images in a face matching task. Highest accuracy was found for conditions using LV target images paired with probe images from the same LV set. Likewise, Honig et al. (2022) report the best performance when participants viewed three highly similar images during the study phase and saw very similar images at test. Neither of these conditions require broad generalisation of learning. Nonetheless, to the extent that people are biased to recognize faces when their appearance resembles that in previous encounters, then experiencing high variability in a face's appearance will allow for better recognition in daily life. In the words of Raviv et al. (2022), exposure to heterogeneity turns the problem of extrapolation into one of interpolation (see also Hasson et al., 2020).

In contrast with Baker et al. (2017), we found no evidence of improved sensitivity after viewing LV images relative to viewing a single image. One difference between our study and Baker et al.'s (Experiment 2) was the number of LV images shown; whereas in our study participants viewed six images, in their study participants viewed ten. It is possible that perceivers require more images (an increase in numerosity) to show significant evidence of learning when variability during the study phase is low. However, both Experiments 1 and 2 of the current study revealed a numerical decrease in sensitivity in the LV vs. single-image condition, suggesting not even a hint of improvement from viewing six LV images. This is consistent with over-fitting that occurs across domains when training sets are very specific, precluding generalisation (Raviv et al., 2022). Another difference between the studies was in the images visible to participants at test. In our study, the LV study images were either visible at test (matching task) or removed (memory); no other reference images were visible to participants. In Baker et al.'s study, participants were shown low-variability images during learning but a reference image that was taken on another day remained visible to participants as they completed the recognition task. Thus, their participants got to view several images of how the identity appeared across one encounter plus a single image of how she appeared in a different encounter. That exposure might have added enough variability to facilitate recognition of the identity in novel images, perhaps by expanding the range of variability anticipated as belonging to the same identity (see Raviv et al., 2022).

A recent study showed that learning a new face in a single live social interaction lasting as little as 5 min does lead to learning (Popva & Wiese, 2023). Such interactions differ from static images in two key ways: They provide rich visual information and are accompanied by biographical information which could help learning. Here we strictly examined whether generalisation of learning occurs when learning is based on viewing static images taken from a single, as compared to multiple, encounters.

Across both experiments, we found a benefit of viewing HV images in the matching and memory versions of our task. Sensitivity was higher in the matching than in the memory task in Experiment 2, but there was no interaction with variability condition in either study. These findings contrast with the results of Ritchie et al. (2021) who tested the utility of exposure to variability in matching tasks where an array of HV or LV images were presented either simultaneously (no memory) or sequentially (requiring memory) with the target image. An overall variability benefit was found only in the sequential task. The authors argued the sequential task requires participants to abstract a representation of the identity and hold that in memory in order to compare to the target image and that this abstraction requirement, which is not present in the simultaneous task, is key to learning faces from variable images. In the present study, we found a variability advantage in both our matching and memory tasks. The difference between our matching task results and those of the simultaneous matching task in Ritchie et al. (2021) may lie in the difference in presentation of training stimuli during the learning phase. In our matching task, images were presented one at a time (i.e., over time) and remained on display during the subsequent recognition test, whereas Ritchie et al. (2021) presented the images in an array simultaneously with the test image. Our sequential presentation of the training images over time may have encouraged the formation of a representation more than matching studies that present multiple training images all at once.

Our data provide novel insights about the process by which a newly encountered face becomes familiar. Face identification is inherently challenging; it requires discriminating match vs. mismatched pairs in a noisy environment where photos of the same person can look very different, and photos of different people can look very similar. Sensitivity to identity might be improved by increasing the perceptual distance between identities and/or by decreasing the perceptual distance between different images of the same identity. Although norm-based coding models emphasize the role of between-identity distance in performance (Leopold et al., 2001; Rhodes & Jeffery, 2006; Valentine, 1991), other evidence suggests that better recognition of familiar (vs. unfamiliar) faces is attributable to a shift in perceived within-identity similarity. White et al. (2022) asked participants to rate the similarity of identity-specific prototypes paired with sex-matched averages (between-identity similarity), and of individual images paired with the same-identity prototype (within-identity similarity). Only within-identity ratings differed as a function of familiarity, such that individual images were rated more similar to the same-identity average when faces were familiar. Baker and Mondloch (2023) examined face recognition as learning unfolds (after participants viewed one, three, six, or nine images of a target identity). Using a Dual Process Signal Detection Model, they provided evidence that learning is associated with an increase in both recollection old (a threshold process reflecting high confident hits) and recollection new (a threshold process reflecting high confidence correct rejections). Their findings suggest that perceptual distances both within and between identities play a role in face learning. The findings of the current study suggest that exposure to high variability in appearance is key to one or both of these processes.

Exposure to high within-person variability in appearance also influenced participants’ response bias; in both experiments, participants were less conservative in the HV condition than in the LV and 1-image control condition. Such a finding provides evidence that face learning involves accepting more images as having sufficient likeness to a target identity to warrant a match response, consistent with increased within-identity similarity. Although not significant, we discovered a numerical difference between the LV and 1-image condition, such that participants tended to be more conservative in the LV condition. Future research should explore this pattern, as it suggests that encountering a face only once constrains the range of variability accepted. Changes in criterion across conditions align with recent calls to conceptualize face recognition not just as a perceptual problem, but also as a decision-making problem (Baker et al., 2023; Bindemann & Burton, 2021).

The importance of exposure to within-person variability in appearance is attributable to the idiosyncratic nature of within-person variability in appearance. Principal Components Analysis of within-person variability reveals that the way in which any given face changes in appearance varies across individuals (Burton et al., 2016); thus, learning one face provides only minimal transfer of learning to the next (but see Strathie et al., 2022). Exposure to idiosyncratic variability (e.g., open-mouth smiles) improves recognition (Mileva & Burton, 2018) and influences perceived similarity. For example, which photos of celebrities are judged as being a good likeness varies across perceivers as a function of which movies each perceiver has seen and degree of familiarity with an identity predicts the likeness ratings given to all photos (Ritchie et al., 2018). Given that perceived within-person variability is idiosyncratic and drives the effect of familiarity on recognition, it is not surprising that between-day variability in appearance—variability that incorporates idiosyncratic variability, is key to face learning. Future research should examine which aspects of between-day variability in appearance (e.g., changes in lighting, camera lens, make-up, and hairstyle) are key to the process by which a face becomes familiar. Evidence from a learning study using the stimuli from Ritchie and Burton (2017) but with the external facial features masked also reported better recognition in the HV versus LV condition (Robins et al., 2018), suggesting that variability in the appearance of internal features is key.

Our findings do not exclude a role for exposure time or sparse social representations (Devue et al., 2019; Schwartz & Yovel, 2019; Shoham et al., 2021; Wiese et al., 2019) contributing to people's fragile representations of lesser-known faces, but they provide compelling evidence that exposure to how a face varies over time is key to face learning. Recognizing a waiter, a new acquaintance, a lecturer, or the perpetrator of a crime is challenging, at least in part, because such individuals have been encountered only once (or, in the case of a lecturer, in a single setting with minimal change in appearance)—an effect that might be enhanced for categories of faces with which the perceiver has minimal experience (e.g., other-race/age faces) or for poor face matchers. Future research should examine the extent to which the benefit of high variability is attributable to building a more representative average (i.e., an average that minimizes errors in identification) versus storing instances that capture a wider range of variability in appearance and the relative contributions of an increase in perceived similarity among images of a learned identity versus an increase in perceived differences between a learned identity and other faces.

### Conclusion

Viewing six images of a newly encountered face promotes face learning, but only when the training images were captured on different days (i.e., incorporate variability in lighting, camera, hairstyle, make-up, health, etc.). Images captured during a single encounter do not promote generalisation of learning, despite incorporating changes in head orientation and facial expression. Our findings have implications in applied settings (e.g., for eye-witness testimony that relies on a single encounter; for use of photos on ID cards or unlocking cell phones) and for models of how humans develop expertise with a novel object.

## Notes

1. *d*' = *z*(H) – *z*(FA). Criterion = –0.5 * (*z*(H) + *z*(FA)). *z*(H) and *z*(F) are the *z* transformations of hits and false alarms. Hit and false alarm rates of 0 or 1 were replaced with 1/2*N* and 1 – 1/(2*N*) respectively, with *N* being the maximum number of hits and false alarms.

## References

[bibr1-03010066241234034] Andrews S. Jenkins R. Cursiter H. Burton A. M. (2015). Telling faces together: Learning new faces through exposure to multiple instances. Quarterly Journal of Experimental Psychology, 68, 2041–2050. 10.1080/17470218.2014.1003949 25607814

[bibr2-03010066241234034] Baker K. A. Laurence S. Mondloch C. J. (2017). How does a newly encountered face become familiar? The effect of within-person variability on adults’ and children’s perception of identity. Cognition, 161, 19–30. 10.1016/j.cognition.2016.12.012 28092773

[bibr3-03010066241234034] Baker K. A. Mondloch C. J. (2019). Two sides of face learning: Improving between-identity discrimination while tolerating more within-person variability in appearance. Perception, 48, 1124–1145. 10.1177/0301006619867862 31483735

[bibr4-03010066241234034] Baker K. A. Mondloch C. J. (2023). Unfamiliar face matching ability predicts the slope of face learning. Scientific Reports, 13, 5248. 10.1038/s41598-023-32244-w 37002382 PMC10066355

[bibr5-03010066241234034] Baker K. A. Stabile V. J. Mondloch C. J. (2023). Stable individual differences in unfamiliar face identification: Evidence from simultaneous and sequential matching tasks. Cognition, 232, 105333. 10.1016/j.cognition.2022.105333 36508992

[bibr6-03010066241234034] Bindemann M. Burton A. M. (2021). Steps towards a cognitive theory of unfamiliar face matching. In M. Bindemann. (Ed.), Forensic face matching (pp. 38–61). Oxford University Press. 10.1093/oso/9780198837749.003.0003

[bibr7-03010066241234034] Bindemann M. Sandford A. (2011). Me, myself, and I: Different recognition rates for three photo-IDs of the same person. Perception, 40, 625–627. 10.1068/p7008 21882725

[bibr8-03010066241234034] Burton A. M. (2013). Why has research in face recognition progressed so slowly? The importance of variability. Quarterly Journal of Experimental Psychology, 66(8), 1467–1485. 10.1080/17470218.2013.800125 23742022

[bibr9-03010066241234034] Burton A. M. Jenkins R. Hancock P. J. B. White D. (2005). Robust representations for face recognition: The power of averages. Cognitive Psychology, 51, 256–284. 10.1016/j.cogpsych.2005.06.003 16198327

[bibr10-03010066241234034] Burton A. M. Kramer R. S. S. Ritchie K. L. Jenkins R. (2016). Identity from variation: Representations of faces derived from multiple instances. Cognitive Science, 40, 202–223. 10.1111/cogs.12231 25824013

[bibr11-03010066241234034] Burton A. M. Wilson S. Cowan M. Bruce V. (1999). Face recognition in poor-quality video: Evidence from security surveillance. Psychological Science, 10, 243–248. 10.1111/1467-9280.00144

[bibr12-03010066241234034] Chen W. Liu C. H. (2009). Transfer between pose and expression training in face recognition. Vision Research, 49, 368–373. 10.1016/j.visres.2008.11.003 19056417

[bibr13-03010066241234034] Devue C. Wride A. Grimshaw G. M. (2019). New insights on real-world human face recognition. Journal of Experimental Psychology General, 148, 994–1007. 10.1037/xge0000493 30247062

[bibr14-03010066241234034] Dowsett A. J. Sandford A. Burton A. M. (2016). Face learning with multiple images leads to fast acquisition of familiarity for specific individuals. Quarterly Journal of Experimental Psychology, 69, 1–10. 10.1080/17470218.2015.1017513 25671778

[bibr15-03010066241234034] Duchaine B. Nakayama K. (2006). The Cambridge face memory test: Results for neurologically intact individuals and an investigation of its validity using inverted face stimuli and prosopagnosic participants. Neuropsychologia, 44, 576–585. 10.1016/j.neuropsychologia.2005.07.001 16169565

[bibr16-03010066241234034] Faul F. Erdfelder E. Lang A.-G. Buchner A. (2007). GPower 3: A flexible statistical power analysis program for the social, behavioral, and biomedical sciences. Behavior Research Methods, 39, 175–191. 10.3758/BF03193146 17695343

[bibr17-03010066241234034] Gonzalez C. Madhavan P. (2011). Diversity during training enhances detection of novel stimuli. Journal of Cognitive Psychology, 23, 342–350. 10.1080/20445911.2011.507187

[bibr18-03010066241234034] Hasson U. Nastase S. A. Goldstein A. (2020). Direct fit to nature: An evolutionary perspective on biological and artificial neural networks. Neuron, 105, 416–434. 10.1016/j.neuron.2019.12.002 32027833 PMC7096172

[bibr19-03010066241234034] Hill H. Schyns P. G. Akamatsu S. (1997). Information and viewpoint dependence in face recognition. Cognition, 62, 201–222. 10.1016/S0010-0277(96)00785-8 9141907

[bibr20-03010066241234034] Hole G. J. George P. A. Eaves K. Rasek A. (2002). Effects of geometric distortions on face-recognition performance. Perception, 31, 1221–1240. 10.1068/p3252 12430949

[bibr21-03010066241234034] Honig T. Shoham A. Yovel G. (2022). Perceptual similarity modulates effects of learning from variability on face recognition. Vision Research, 201, 108128–108128. 10.1016/j.visres.2022.108128 36272208

[bibr22-03010066241234034] Hussain Z. Bennett P. J. Sekuler A. B. (2012). Versatile perceptual learning of textures after variable exposures. Vision Research, 61, 89–94. 10.1016/j.visres.2012.01.005 22266193

[bibr23-03010066241234034] JASP Team. (2020). *JASP* (Version 0.14.0) [Computer software].

[bibr24-03010066241234034] Jenkins R. White D. Van Montfort X. Mike Burton A. (2011). Variability in photos of the same face. Cognition, 121, 313–323. 10.1016/j.cognition.2011.08.001 21890124

[bibr25-03010066241234034] Kramer R. Ritchie K. L. Burton M. A. (2015). Viewers extract the mean from images of the same person: A route to face learning. Journal of Vision, 15, 1–9. 10.1167/15.4.1 26067175

[bibr26-03010066241234034] Lander K. Bruce V. (2003). The role of motion in learning new faces. Visual Cognition, 10, 897–912. 10.1080/13506280344000149

[bibr27-03010066241234034] Laurence S. Zhou X. Mondloch C. J. (2016). The flip side of the other-race coin: They all look different to me. British Journal of Psychology, 107, 374–388. 10.1111/bjop.12147 26366460

[bibr28-03010066241234034] Leopold D. A. O’Toole A. J. Vetter T. Blanz V. (2001). Prototype-referenced shape encoding revealed by high-level aftereffects. Nature Neuroscience, 4, 89–94. 10.1038/82947 11135650

[bibr29-03010066241234034] Matthews C. M. Davis E. E. Mondloch C. J. (2018). Getting to know you: The development of mechanisms underlying face learning. Journal of Experimental Child Psychology, 167, 295–313. 10.1016/j.jecp.2017.10.012 29220715

[bibr30-03010066241234034] Matthews C. M. Mondloch C. J. (2018a). Finding an unfamiliar face in a line-up: Viewing multiple images of the target is beneficial on target-present trials but costly on target-absent trials. British Journal of Psychology, 109, 758–776. 10.1111/bjop.12301 29658990

[bibr31-03010066241234034] Matthews C. M. Mondloch C. J. (2018b). Improving identity matching of newly encountered faces: Effects of multi-image training. Journal of Applied Research in Memory and Cognition, 7, 280–290. 10.1016/j.jarmac.2017.10.005

[bibr32-03010066241234034] Matthews W. J. Benjamin C. Osborne C. (2007). Memory for moving and static images. Psychonomic Bulletin & Review, 14, 989–993. 10.3758/BF03194133 18087971

[bibr33-03010066241234034] Megreya A. M. Sandford A. Burton A. M. (2013). Matching face images taken on the same day or months apart: The limitations of photo ID: Matching face images. Applied Cognitive Psychology, 27, 700–706. 10.1002/acp.2965

[bibr34-03010066241234034] Menon N. White D. Kemp R. I. (2015). Identity-level representations affect unfamiliar face matching performance in sequential but not simultaneous tasks. Quarterly Journal of Experimental Psychology, 68, 1777–1793. 10.1080/17470218.2014.990468 25686094

[bibr35-03010066241234034] Mileva M. Burton A. M. (2018). Smiles in face matching: Idiosyncratic information revealed through a smile improves unfamiliar face matching performance. The British Journal of Psychology, 109, 799–811. 10.1111/bjop.12318 29920996

[bibr36-03010066241234034] Murphy J. Ipser A. Gaigg S. B. Cook R. (2015). Exemplar variance supports robust learning of facial identity. Journal of Experimental Psychology. Human Perception and Performance, 41, 577–581. 10.1037/xhp0000049 25867504 PMC4445380

[bibr37-03010066241234034] Nordt M. Weigelt S. (2017). Face recognition is similarly affected by viewpoint in school-aged children and adults. PeerJ, e3253. 10.7717/peerj.3253 PMC5438580 28533951

[bibr38-03010066241234034] Noyes E. Jenkins R. (2019). Deliberate disguise in face identification. Journal of Experimental Psychology. Applied, 25, 280–290. 10.1037/xap0000213 30730157

[bibr39-03010066241234034] Popva T. Wiese H. (2023). How quickly do we learn new faces in everyday life? Neurophysiological evidence for face identity learning after a brief real-life encounter. Cortex, 159, 205–216. 10.1016/j.cortex.2022.12.005 36640620

[bibr40-03010066241234034] Raviv L. Lupyan G. Green S. C. (2022). How variability shapes learning and generalization. Trends in Cognitive Sciences, 26, 462–483. 10.1016/j.tics.2022.03.007 35577719

[bibr41-03010066241234034] Rhodes G. Jeffery L. (2006). Adaptive norm-based coding of facial identity. Vision Research, 46, 2977–2987. 10.1016/j.visres.2006.03.002 16647736

[bibr42-03010066241234034] Ritchie K. L. Burton A. M. (2017). Learning faces from variability. Quarterly Journal of Experimental Psychology, 70, 897–905. 10.1080/17470218.2015.1136656 26831280

[bibr43-03010066241234034] Ritchie K. L. Kramer R. S. Burton A. M. (2018). What makes a face photo a “good likeness”? Cognition, 170, 1–8. 10.1016/j.cognition.2017.09.001 28917125

[bibr44-03010066241234034] Ritchie K. L. Kramer R. S. Mileva M. Sandford A. Burton A. M. (2021). Multiple-image arrays in face matching tasks with and without memory. Cognition, 211, 1–11. 10.1016/j.cognition.2021.104632 33621739

[bibr45-03010066241234034] Ritchie K. L. Mireku M. O. Kramer R. S. (2020). Face averages and multiple images in a live matching task. British Journal of Psychology, 111, 92–102. 10.1111/bjop.12388 30945267

[bibr46-03010066241234034] Robins E. Susilo T. Ritchie K. Devue C. (2018). Within-person variability promotes learning of internal facial features and facilitates perceptual discrimination and memory. 10.31219/osf.io/5scnm.

[bibr47-03010066241234034] Sandford A. Ritchie K. L. (2021). Unfamiliar face matching, within-person variability, and multiple-image arrays. Visual Cognition, 29, 143–157. 10.1080/13506285.2021.1883170 33621739

[bibr48-03010066241234034] Schwartz L. Yovel G. (2019). Independent contribution of perceptual experience and social cognition to face recognition. Cognition, 183, 131–138. 10.1016/j.cognition.2018.11.003 30448534

[bibr49-03010066241234034] Shoham A. Kliger L. Yovel G. (2021). Learning faces as concepts improves face recognition by engaging the social brain network. Social Cognitive and Affective Neuroscience, 17, 290–299. 10.1093/scan/nsab096 34402904 PMC8881637

[bibr50-03010066241234034] Strathie A. Hughes-White N. Laurence S. (2022). The sibling familiarity effect: Is within-person facial variability shared across siblings? British Journal of Psychology, 113, 327–345. 10.1111/bjop.12517 34232512

[bibr51-03010066241234034] Troje N. F. Bülthoff H. H. (1996). Face recognition under varying poses: The role of texture and shape. Vision Research (Oxford), 36, 1761–1771. 10.1016/0042-6989(95)00230-8 8759445

[bibr52-03010066241234034] Valentine T. (1991). A unified account of the effects of distinctiveness, inversion, and race in face recognition. The Quarterly Journal of Experimental Psychology, 43, 161–204. 10.1080/14640749108400966 1866456

[bibr53-03010066241234034] Wallraven C. Schwaninger A. Schumacher S. Bülthoff H. H. (2002). View-based recognition of faces in man and machine: Re-visiting inter-extra-ortho. Lecture Notes in Computer Science, 2525, 651–660.

[bibr54-03010066241234034] Watson T. L. Robbins R. A. Best C. T. (2014). Infant perceptual development for faces and spoken words: An integrated approach. Developmental Psychobiology, 56, 1454–1481. 10.1002/dev.21243 25132626 PMC4231232

[bibr55-03010066241234034] Wetzels R. Matzke D. Lee M. Rouder J. Iverson G. Wagenmkers E. (2011). Statistical evidence in experimental psychology. Perspectives on Psychological Science, 6(3), 291–298. 10.1177/1745691611406923 26168519

[bibr56-03010066241234034] White D. Kemp R. I. Jenkins R. Matheson M. Burton A. M. (2014). Passport officers’ errors in face matching. PloS One, 9, 1–6. 10.1371/journal.pone.0103510 PMC4136722 25133682

[bibr57-03010066241234034] White D. Wayne T. Varela V. P. L. (2022). Partitioning natural face image variability emphasises within-identity over between-identity representation for understanding accurate recognition. Cognition, 219, 104966–104966. 10.1016/j.cognition.2021.104966 34861575

[bibr58-03010066241234034] Wiese J. Tüttenberg S. C. Ingram B. T. Chan C. Y. X. Gurbuz Z. Burton A. M. Young A. W. (2019). A robust neural index of high face familiarity. Psychological Science, 30, 261–272. 10.1177/0956797618813572 30557087

[bibr59-03010066241234034] Young A. W. Burton A. M. (2017). Recognizing faces. Current Directions in Psychological Science, 26, 212–217. 10.1177/0963721416688114

[bibr60-03010066241234034] Zhou X. Mondloch C. J. Chien S. H. L. Moulson M. C. (2022). Multi-cultural cities reduce disadvantages in recognizing naturalistic images of other-race faces: Evidence from a novel face learning task. Scientific Reports, 12, 1–14. 10.1038/s41598-022-11550-9 35624118 PMC9142532

